# Rationalism in Medical Treatment; or, the Restoration of Chemism, the System of the Future

**Published:** 1885-12

**Authors:** 


**Rationalism in Medical Treatment; or, The Restoration of Chemism, the System of the Future.** By WILLIAM THORNTON. Published by the author. 46 pp.; 12 mo.

This is a very small book with a very pretentious title, published by the author as an advertising venture. The old cry that the regular profession is intolerant of all progress, is raised to show why the writer has not been honored and knighted as a reward for his remarkable discoveries (?). The work of the printer is to be especially commended.

